# Gaussian Modelling Characteristics of Peripheral Arterial Pulse: Difference between Measurements from the Three Trimesters of Healthy Pregnancy

**DOI:** 10.1155/2018/1308419

**Published:** 2018-10-11

**Authors:** Kunyan Li, Song Zhang, Lin Yang, Hongqing Jiang, Dongmei Hao, Lei Zhang, Dingchang Zheng

**Affiliations:** ^1^College of Life Science and Bioengineering, Beijing University of Technology, Beijing 100124, China; ^2^Department of Medical Science and Public Health, Faculty of Medical Science, Anglia Ruskin University, Chelmsford CM1 1SQ, UK; ^3^Haidian Maternal & Child Health Hospital, Beijing 100026, China

## Abstract

Arterial pulse wave analysis has been attempted to monitor the maternal physiological changes of circulatory system during pregnancy. This study aimed to quantify the difference of Gaussian modelling characteristics derived from radial pulses measured from the three trimesters of healthy pregnant women. Radial pulses were recorded from seventy pregnant women between gestational week 11–13, week 20–22, and then week 37–39. They were then normalized and decomposed into three independent Gaussian waves for deriving four key modelling characteristic parameters: including the peak time interval (*T*) and peak amplitude ratio (*R*) between the first and second Gaussian waves (*T*_1,2_ and *R*_1,2_), and their corresponding values between the first and third Gaussian waves (*T*_1,3_ and *R*_1,3_). Post hoc multiple comparisons after analysis of variance was then applied to study the within-subject differences in Gaussian modelling characteristics between the three trimesters. The key results were that *T*_1,2_ and *T*_1,3_ increased significantly (*T*_1,2_: 12.8 ± 1.3 vs 13.2 ± 1.3, *p* < 0.05; *T*_1,3_: 39.5 ± 4.3 vs 45.4 ± 5.1, *p* < 0.001), and *R*_1,3_ decreased significantly from the first to second trimester (0.60 ± 0.15 vs 0.53 ± 0.11, *p* < 0.001). From the second to third trimester, *T*_1,2_ decreased significantly (13.2 ± 1.3 vs 12.8 ± 1.2, *p* < 0.01), and *T*_1,3_ and *R*_1,3_ decreased slightly but nonsignificantly. Since larger *T*_1,2_ and *T*_1,3_ and smaller *R*_1,3_ are associated with more compliant peripheral arteries, our results indicated that peripheral arteries become more compliant from the first to second trimester and then have a tendency of returning to baseline during normal pregnancy. In conclusion, this study has quantitatively demonstrated significant changes of Gaussian modelling characteristics derived from radial pulses at the three trimesters of normal pregnant women, suggesting that these modelling characteristics could be used as parameters in monitoring maternal physiological changes during normal pregnancy.

## 1. Introduction

The maternal physiological changes of circulatory system during pregnancy are essential to support and protect the development of fetus [1, 2]. The cardiovascular system of pregnant women adapts to the increased cardiac demand. Increased cardiac output has been observed at the early stage of pregnancy with increased stroke volume and heart rate (HR). As pregnancy progresses, the increased stoke volume reaches a plateau state but HR continues to increase. In addition, it has been reported that systemic vascular resistance decreases during normal pregnancy [3, 4].

Arterial pulse wave analysis has been attempted to assess maternal physiological changes since arterial pulses contains useful physiological information about cardiovascular system [5–8]. The commonly used indices include the carotid-radial pulse wave velocity (crPWV), augmentation index (AIx), or AIx at heart rate of 75/min (AIx-75) derived from carotid arterial pulses [9–12]. Their longitudinal changes during normal pregnancy and their differences between normotensive and hypertensive pregnant women have been quantified [13–15]. Although different arterial pulse wave analysis techniques have been employed in these published studies, a similar conclusion has been drawn, which was that the carotid arteries become more compliant at the early stage of pregnancy, then return to baseline at the late stage of pregnancy. There were also a few studies to investigate the maternal physiological changes during normal pregnancy using peripheral arterial pulses. Oyama-Kato et al. and Gomez et al. used pulse wave velocity (PWV) measured from brachial artery and concluded that peripheral arterial compliance increased at the early stage of pregnancy and then returned to baseline at late pregnancy [16, 17]. In order to obtain the arterial pulse waveform characteristics, the published studies mainly used derivatives methods. The main disadvantage of using derivatives methods is that they were quite sensitive to noise, and its measurement reliability was not good enough especially when there were no obvious tidal or dicrotic points on the arterial pulses [18].

In order to overcome these difficulties, Gaussian modelling of arterial pulses has been proposed, which can decompose the arterial pulse waveform into different individual Gaussian waves [19]. Liu et al. reported that both carotid and radial arterial pressure pulse waveforms could be accurately modelled using three Gaussian functions, which were considered to be associated with forward wave, the tide, and dicrotic or backward waves [20]. Several Gaussian modelling characteristics, including the peak amplitude, the peak time positions, and half-width of Gaussian waves, have been extracted from each Gaussian function to reflect the physiological changes of peripheral circulatory system. Rubins applied Gaussian modelling methods to simultaneously measured finger and ear photoplethymography (PPG) pulses to evaluate the ageing effect on the Gaussian modelling characteristics [18]. The differences in Gaussian modelling characteristics between healthy subjects and heart failure patients and during exercise and recovery have also been investigated to explore its potential clinical applications [21, 22]. However, to the best of our knowledge, there is no study to quantify the changes of Gaussian modelling characteristics during normal pregnancy.

This study aimed to comprehensively quantify the changes of Gaussian modelling characteristics derived from radial pulses measured from the three trimesters of normal pregnancy.

## 2. Materials and Methods

### 2.1. Subjects

Seventy healthy pregnant women volunteers, with average age of 31 ± 4 years, height of 162 ± 6 cm, weight of 56 ± 4 kg, and body mass index of 21 ± 2 kg/m^2^, were recruited at the Haidian Maternal and Child Health Hospital, Beijing. Pregnant women with multiple pregnancies, abnormal menstrual cycle, chronic hypertension, diabetes, anemia, and any other known diseases during pregnancy were excluded from this study.

The institutional review board of Beijing University of Technology approved the study, which was conducted in compliance with the Declaration of Helsinki. After each individual volunteer read the Participant Consent Form, understood the purpose of this study, and agreed to take part in, a written informed consent was obtained. Before the experiments, all the volunteers were abstained from alcohol, caffeine, and any drugs.

### 2.2. Radial Pulse Measurement

Radial pulses were measured from each pregnant woman at three different stages of normal pregnancy: the early pregnancy (the first trimester between week 11–13), middle pregnancy (the second trimester between week 20–22), and late pregnancy (the third trimester between week 37–39). This is referred to the guidelines of American Congress of Obstetricians and Gynecologists [23]. All pregnant women were required to attend the three visits for radial pulse measurements at the above three gestational weeks.

The arterial pulse measurements were performed in a quiet clinical measurement room at the Haidian Maternal and Child Health Hospital, Beijing, China. During each of the three visits, all the pregnant women were firstly asked to sit on a chair quietly for five minutes to achieve stable cardiovascular status before formal arterial pulse recording. Resting systolic and diastolic blood pressures (SBP and DBP) and HR were then measured using a validated electronic sphygmomanometer (HEM-7124 from Omron Crop). With the pregnant woman in a supine position, a pressure sensor (MB-3) was placed on the left wrist above the radial artery, where was marked, to record analyzable radial pulses for three minutes using a PowerLab data collection system (ADInstrumrnts Pty Ltd., PowerLab 8/35, Bella Vista NSW 2153, Australia) at a sampling rate of 1000 Hz for three times. Univariate ANOVA analyses showed that there were no significant differences between the three repeated measurements (all *p* > 0.05), demonstrating the reliability of this experiment. Therefore, their average values from the three repeated measurements were calculated as reference values for each subject, which were used for further statistical analysis. Therefore, there were three radial pulse recordings from each of the seventy pregnant women.

### 2.3. Gaussian Modelling of Radial Pulse

**Figure 1:**
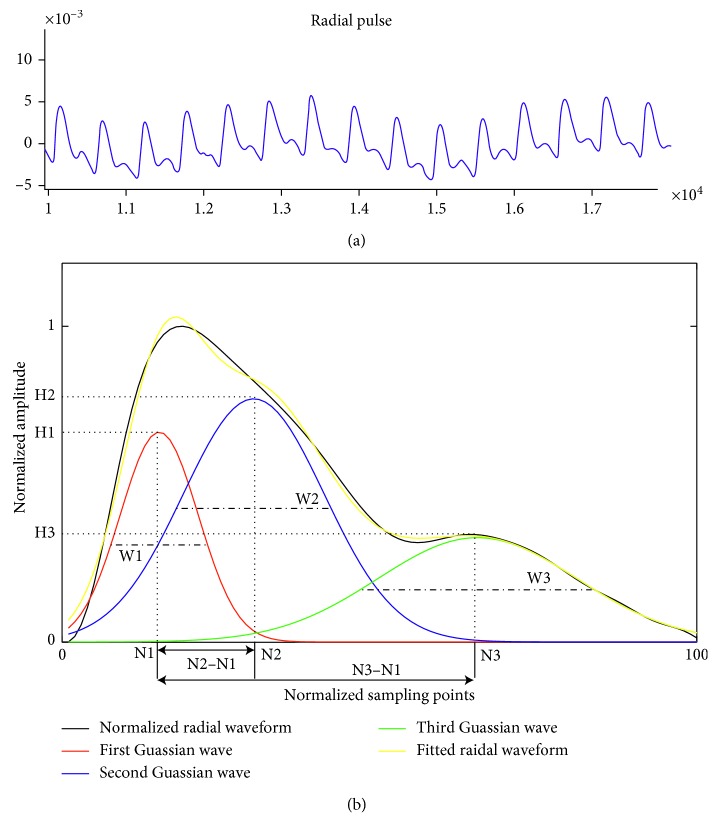
Demonstration of Gaussian modelling characteristics derived from orginal radial pulse waveform. (a) A segment of the original arterial pulse waveform measured by the pressure sensor; (b) Gaussian modelling of a radial pulse waveform. The radial pulse waveform was decomposed into three positive Gaussian waves as shown in red, blue, and green. The yellow curve shows the Gaussian fitted radial waveform.

All the recorded radial pulses were firstly processed to remove baseline drift. They were then normalized in both amplitude and width, and then decomposed into three positive Gaussian functions *f*_*i*_(*n*), *i*=1,2,3, as proposed by Liu et al. [20]. The first Gaussian function *f*_1_(*n*) denotes the main wave, or the forward wave; the second Gaussian function *f*_2_(*n*) denotes the tidal wave; and the third Gaussian function *f*_3_(*n*) denotes the dicrotic wave, or the backward wave. Each Gaussian function was defined as

(1) $$\begin{array}{c} f_{i}\left(n\right)=H_{i}\times e^{\left(-2{\left(n-N_{i}\right)}^{2}/W_{i}^{2}\right)},\;i=1,2,3;\;\;n=1,2,\ldots ,100, \end{array}$$

where *H*_*i*_ means the peak amplitude, *N*_*i*_ means the peak time position, and *W*_*i*_ means the half-width of each Gaussian wave, as shown in Figure 1. The acquired curve as show in black, the yellow curve shows the Gaussian fitted radial waveform. There is no significant difference between the acquired curve and fitted curve (*p* > 0.05).

### 2.4. Determination of Gaussian Modelling Characteristics of Radial Arterial Pulse

Nine basic Gaussian modelling characteristics, including the peak amplitude (H_1_, H_2_, H_3_), peak time position (*N*_1_, *N*_2_, *N*_3_), and half-width (*W*_1_, *W*_2_, *W*_3_), were derived from the three Gaussian curves, as shown in Figure 1. Four extended modelling characteristics were also determined, including the peak time interval (*T*_1,2_, *T*_1,3_) and amplitude ratio (*R*_1,2_, *R*_1,3_) between first and second Gaussian waves and between the first and third Gaussian waves, which were calculated as

(2) $$\begin{array}{c} T_{1,2}=N_{2}-N_{1};\\ T_{1,3}=N_{3}-N_{1},\\ R_{1,2}=\frac{H_{2}}{H_{1}};\\ R_{1,3}=\frac{H_{3}}{H_{1}}. \end{array}$$

### 2.5. Data and Statistical Analysis

The means ± SDs of blood pressure (BP), HR, and all the derived Gaussian modelling characteristics (*H*_1_, *H*_2_, *H*_3_, *N*_1_, *N*_2_, *N*_3_, *W*_1_, *W*_2_, *W*_3_, *T*_1,2_, *T*_1,3_, *R*_1,2_, *R*_1,3_) were calculated across all the pregnant women, separately for the three trimesters. Analysis of variance and post hoc multiple comparisons were then performed using SPSS (Version 20.0; SPSS Inc., Chicago, IL, USA) to investigate the effect of the three trimesters on all the Gaussian modelling characteristics and to compare whether there were significant within-subject differences between the three trimesters during pregnancy. A *p* < 0.05 was used as the significant criterion.

## 3. Results and Discussion

### 3.1. Changes of Heart Rate and Blood Pressures at Three Trimesters

As shown in Table 1, HR increased significantly, and BPs (SBP and DBP) decreased significantly during normal pregnancy (all *p* < 0.01 with the comparisons between the first and second trimesters and between the first and third trimesters).

### 3.2. Gaussian Wave Peak, Height, and Half-Width between the Three Trimesters

**Figure 2:**
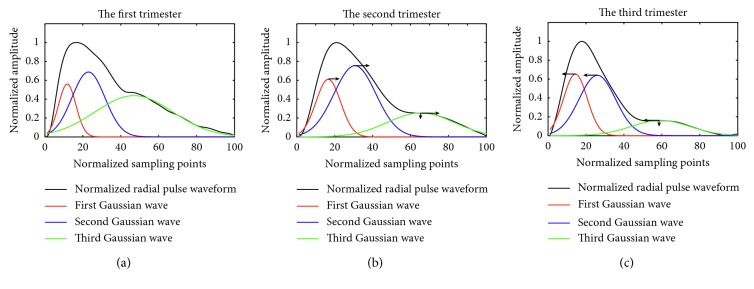
Illustration of Gaussian modelling of normalized radial pulse waveform at the first (a), second (b), and third (c) trimesters of a pregnant woman. The three Gaussian waves are shown in red, blue, and green. The arrows show the changes of Gaussian peak time position and peak amplitude.

Figure 2 gives one example of the Gaussian modelling of a normalized radial pulse waveform at the three trimesters of a pregnant woman. It illustrates that the peak time positions of all the three Gaussian waves moved toward right from the first to the second trimester, and then moved backward at the third trimester. The peak amplitude of third Gaussian wave decreased continuously at the second and third trimesters in comparison with the first trimester.

**Figure 3:**
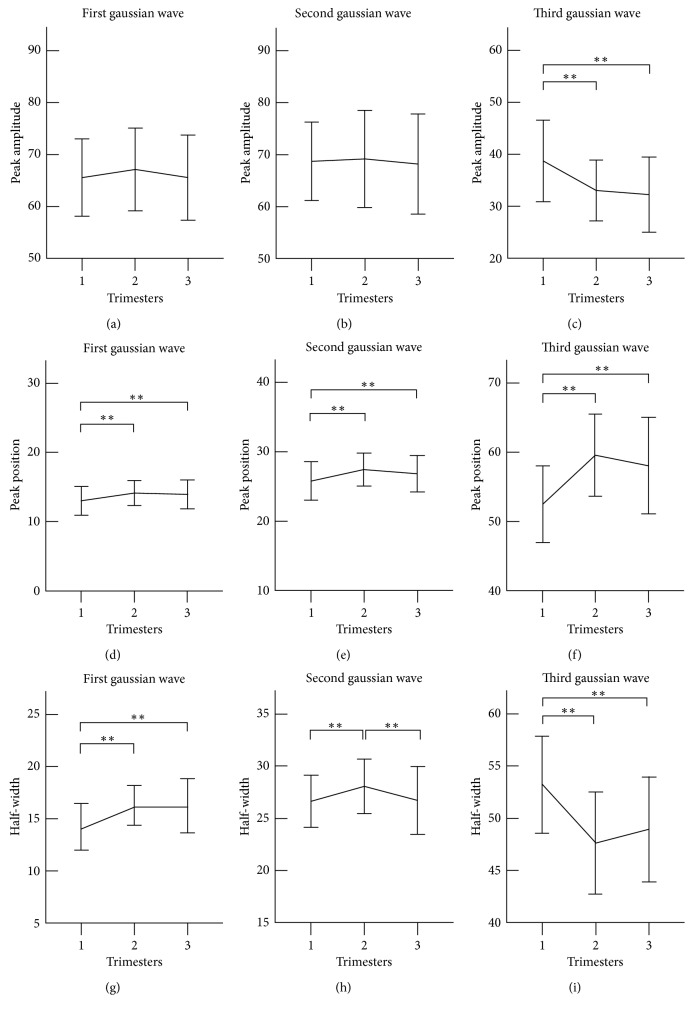
Gaussian modelling characteristics of the radial pulse measured at the three trimesters of healthy pregnant women. Their means ± SDs are given. (a–c) show peak amplitude changes of the first, second, and third Gaussian waves (*H*_1_, *H*_2_, *H*_3_) at the three trimesters; (d–f) show changes of peak time position of the first, second, and third Gaussian waves (*N*_1_, *N*_2_, *N*_3_) at the three trimesters; (g–i) shows changes of half-width of the first, second, and third Gaussian waves (*W*_1_, *W*_2_, *W*_3_) at the three trimesters. ∗∗ represents *p* < 0.01.

Figure 3 shows the changes of the nine basic Gaussian modelling characteristics at the three trimesters. It can be observed from the first Gaussian wave that its peak time position and half-width had a similar changing trend. They significantly increased from the first to second trimester (*N*_1_: 13.0 ± 2.1 vs 14.2 ± 1.8, *W*_1_: 14.2 ± 2.2 vs 16.3 ± 1.9, both *p* < 0.001) and then decreased slightly but nonsignificantly towards baseline at the third trimester. The peak amplitude had a similar changing trend at the peak time position and half-width, but there was no significant difference between any two trimesters (all *p* > 0.05).

The three basic modelling characteristics from the second Gaussian wave changed in a similar pattern as those from the first Gaussian wave. The peak time position significantly increased from the first to second trimester (*N*_2_: 25.8 ± 2.8 vs 27.5 ± 2.4, *p* < 0.01), then decreased slightly but nonsignificantly towards baseline at the third trimester. The half-width increased significantly from the first to second trimester (*W*_2_: 26.6 ± 2.5 vs 28.1 ± 2.6, *p* < 0.01), then decreased significantly to return to baseline from the second to third trimester (*W*_2_: 28.1 ± 2.6 vs 26.7 ± 3.2, *p* < 0.01).

Regarding the modelling characteristics from the third Gaussian wave, there were significant differences between the first and second and between first and third trimesters for the three characteristics (all *p* < 0.001), but not between second and third trimesters. In detail, the peak amplitude and the half-width of the third Gaussian wave decreased significantly, and the peak time position increased significantly (*H*_3_: 38.7 ± 7.8 vs 33.1 ± 5.8; *W*_3_: 53.2 ± 4.6 vs 47.6 ± 4.9; *N*_3_: 52.5 ± 5.5 vs 59.6 ± 5.9; all *p* < 0.001) from the first to second trimester.

### 3.3. Gaussian Wave Peak Time Interval at the Three Trimesters

**Figure 4:**
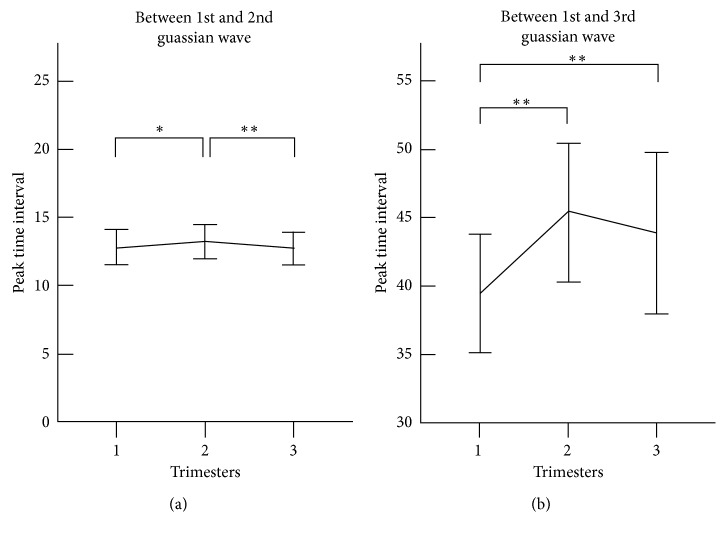
Peak time interval derived from the three Gaussian waves of the radial pulses measured at the three trimesters of healthy pregnant women. Their means ± SDs are given. (a) Changes of peak time interval between first and second Gaussian waves at the three trimesters; (b) Changes of peak time interval between first and third Gaussian waves at the three trimesters. ∗ represents *p* < 0.05, and ∗∗ represents *p* < 0.01.

Figure 4 shows the Gaussian peak time interval derived from the three Gaussian waves of the radial pulses measured at the three trimesters of healthy pregnant women. The peak time interval (*T*_1,2_) between the first and second Gaussian waves increased significantly from the first to second trimester (12.8 ± 1.3 vs 13.2 ± 1.3, *p* < 0.05) and then decreased significantly from the second to third trimester (13.2 ± 1.3 vs 12.8 ± 1.2, *p* < 0.01). The peak time interval between the first and third Gaussian waves (*T*_1,3_) also increased significantly (39.5 ± 4.3 vs 45.4 ± 5.1, *p* < 0.001) and then decreased slightly at the third trimester to return to the baseline.

### 3.4. Gaussian Wave Amplitude Ratio at the Three Trimesters

**Figure 5:**
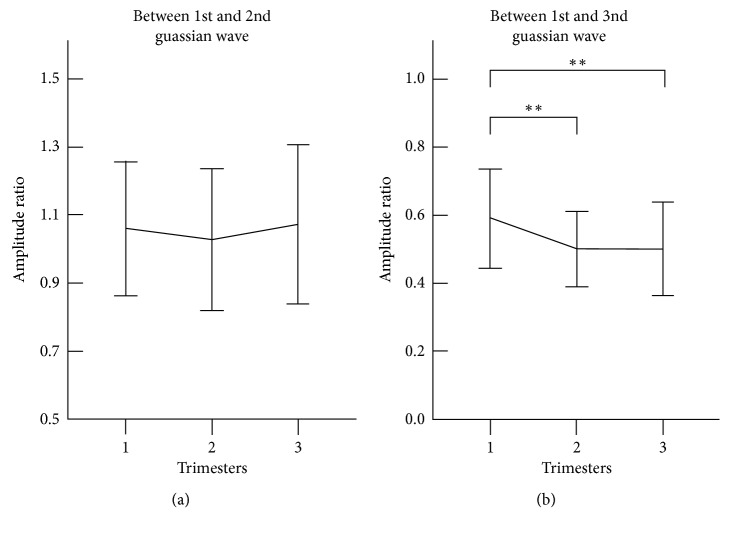
Gaussian amplitude ratio derived from the three Gaussian waves of the radial pulses measured at the three trimesters. Their means ± SDs are given. (a) Changes of amplitude ratio between first and second Gaussian waves at the three trimesters; (b) Changes of amplitude ratio between first and third Gaussian wave at the three trimesters. ∗∗ represents *p* < 0.01.

Figure 5 shows the Gaussian amplitude ratio derived from the three Gaussian waves of the radial pulses measured at the three trimesters of healthy pregnant women. The significant finding is that the amplitude ratio (*R*_1,3_) between the first and third Gaussian waves decreased significantly from the first to second trimester (0.60 ± 0.15 vs 0.53 ± 0.11, *p* < 0.001) and then kept a stable level until the third trimester.

## 4. Discussion

In this study, Gaussian modelling characteristics derived from normalized radial pulses have been quantified and compared between the three trimesters during normal pregnancy. To the best of our knowledge, this is the first study to comprehensively investigate the peripheral arterial pulse waveform changes during normal pregnancy using Gaussian modelling approach.

The first observation of this study with regard to the maternal HR and BPs changes during pregnancy agreed with published studies [24, 25]. HR increased significantly, and BPs decreased significantly from the first to second trimester of normal healthy pregnancy. However, it is noted that it is inappropriate to use the automatic BP monitor to measure BPs in pregnant women for clinical diagnosis.

The key results of this study were about the arterial pulse waveform changes during normal pregnancy. It is known that the arterial pulse waveform is a composite of the forward wave and reflects waves from periphery and that the forward wave or the first Gaussian wave is associated with the ventricular contraction [26]. During pregnancy, an increase in oxygen and nutrients demand requires increased cardiac function of pregnant women, including cardiac output, HR, and stroke volume [1, 27, 28]. This has been evidenced in the study with increased peak time position and the half-width of the first Gaussian wave from the first to second trimester during normal pregnancy.

The second and third Gaussian waves are associated with the reflection from the periphery mainly at branch points, which can be used to quantify the properties of the peripheral arteries [29]. Some published studies have reported that the vascular resistance of peripheral arteries decreases during normal pregnancy [30, 31]. The previous study reported how arterial pulse waveform characteristics changed with lower peripheral resistance, where a narrow and high main wave, unnoticeable tidal wave, highlighted dicrotic wave, and decreased PWV were observed [32]. The decreased PWV causes the tidal or dicrotic waves to move backward further, resulting in increased *N*_2_ and *N*_3_ [22]. In this study, N_2_ and N_3_ increased at early pregnancy and then returned towards baseline. This agreed with Oyama et al.'s study, where it has been reported that the peripheral resistance was reduced firstly and then enhanced during normal pregnancy [16]. It merits further evaluation of the significance of these three constituent waves and the mechanisms that act on them. This could provide evidence that these differences are justified during pregnancy.

It has been explained in the previous studies that the peak time intervals *T*_1,2_ and *T*_1,3_ between different Gaussian waves are associated with the compliance of peripheral arteries [19]. It has also been reported that the amplitude ratios *R*_1,2_ and *R*_1,3_ are related to the AI and reflection index (RI) of peripheral arterial pulses which are two arterial pulse waveform indices commonly used to quantify peripheral arterial stiffness [18, 19, 33, 34]. In general, the larger the *T*_1,2_ and *T*_1,3_ are, or the lower the *R*_1,3_ are, more compliant the peripheral arteries is [19]. The stiffness of carotid arteries of pregnant women has been studied during normal pregnancy using other measurement techniques including PWV, where they concluded that arterial compliance of carotid arteries increased significantly from the first to the second trimester, and then decreased toward baseline at the third trimester [16, 35]. Similar findings have been observed in this study with increased peak time intervals *T*_1,2_ and *T*_1,3_ and decreased amplitude ratios *R*_1,2_ and *R*_1,3_ from the first to the second trimester of normal pregnancy, indicating that maternal peripheral arterial compliance increases from the early to middle stage of pregnancy.

It is also noted that, from the second to third trimester, the Gaussian modelling characteristics of peripheral arterial pulses had a tendency of returning towards baseline values although these changes were not significant for the majority of Gaussian modelling characteristics in this study. Our results were different from those significant changes of cfPWV and AIx derived from aortic reported between the second the third trimesters [13, 16, 36, 37]. These studies demonstrated that indexes derived from central arteries significantly change between the second and third trimesters. Khalil et al. and Fujime et al. reported that central aortic AIx-75 decreased firstly and reached a nadir at the middle phase of pregnancy, then significantly increased at the late stage of pregnancy [13, 36], indicating that the aortic elasticity becomes better and then worse during normal pregnancy. Similar changing pattern in both carotid PWV and brachial-ankle PWV was also observed during normal pregnancy by Yuan et al. and Oyama-Kato et al. [16, 37]. This could be partially explained by the different indices used from different techniques or the different measurement sites along the arterial trees or the different comparisons between different gestational weeks. Therefore, a comprehensive investigation to understand the different changes of these indirect measurements of arterial stiffness at late stage of pregnancy should be conducted in future studies.

There were some limitations to this study. First, even though the same trained operator have been used during the whole experiments, the contact pressure still is difficult to control. Pulse contour could be affected. Moreover, the wave propagation could be related to heart rate and blood pressure. However, it is worth further investigating how the changes in heart rate and blood pressure influence the Gaussian modelling parameters.

## 5. Conclusions

In conclusion, this study has quantitatively demonstrated significant changes of Gaussian modelling characteristics derived from radial pulses at the three trimesters of normal pregnant women. This indicates that peripheral arteries become more compliant from the first to second trimester and then have a tendency of returning to baseline during normal pregnancy and suggests that these modelling characteristics could be used as parameters in monitoring maternal physiological changes during normal pregnancy.

## Figures and Tables

**Table 1:** Means ± SDs of heart rate and blood pressures measured at the three trimesters of normal pregnancy.

Trimesters	HR (beats/min)	SBP (mmHg)	DBP (mmHg)
First	85 ± 12	115 ± 10	73 ± 9
Second	93 ± 11^*∗∗*^	111 ± 8^*∗∗*^	70 ± 7^*∗∗*^
Third	94 ± 13^*∗∗*^	109 ± 11^*∗∗*^	69 ± 9^*∗∗*^

^*∗∗*^ *p* < 0.01 in comparison with the first trimester.

## Data Availability

All data used to support the finding of this study are available from the corresponding author upon request.
